# 

**Published:** 1791

**Authors:** 


### CONTENTS.

Page

I. *A Case of Hydrophobia; with the Appearances on Dissection. By* John Ferriar, *M. D. Physician to the Infirmary at Manchester.* — — — 1

II. *Some Observations on the Prevention and Treatment of Hydrophobia. By Mr.* William Loftie, *Surgeon at Canterbury.* 11

III. *An Account of an uncommon Inflammation of the Epiglottis. By Mr.* Thomas Mainwaring, *Apothecary in London.* — 40

IV. *Cases of the Extraction of the Cataract; with practical Remarks. By Mr.* Richard Sparrow, *one of the Surgeons to the Charitable Infirmary, Dublin; and Member of the Royal College of Surgeons in Ireland.* — — — — 43

V. *Account of an Extra Uterine Conception. By Mr.* William Baynham, *Member of the Corporation of Surgeons of London; and Surgeon in Essex County in Virginia.* 73

Page

VI. *A Case of Spontaneous Evolution of the Fœtus. By Mr.* Richard Simmons, *one of the Surgeons of the British Lying-in Hospital in London.* — — 76

VII. *A Case of Petechiæ sine Febre. By* Samuel Ferris, *M. D. F. A. S. Physician in London.* — — — 79

VIII. *Instance of a Disease, to which* Sauvages *has given the Name of Meteorismus Ventriculi; with Remarks. By* Robert Graves, *M. D. Physician at Sherborne, in Dorsetshire; and Extra Licentiate of the College of Physicians, London.* — — 90

IX. *Case of a Catheter, left in the Bladder, in drawing off the Urine, for a retroversion of the Uterus. By Mr.* Edward Ford, *Surgeon of the Westminster General Dispensary.* — — — — 96

X. *Case of an Imperforate Rectum. By the same.* — — — — 102

XI. *Facts relative to Pemphigus. By Mr.* R. B. Blagden, *Surgeon at Petworth in Sussex.* — — — — 105

XII. *Account of a Fact relative to Menstruation, not hitherto described. By* Thomas Denman, *M. D. Licentiate in Midwifery*

of

Page

*of the Royal College of Physicians, London.* — — — — 108

XIII. *Practical Observations on the Treatment and Causes of the Dropsy of the Brain. By* Thomas Percival, *M. D. F. R. S. and S. A. Lond.; F. R. S. and R. M. S. Edinb.; President of the Literary and Philosophical Society of Manchester; Member of the Royal Medical Society at Paris; of the Royal Society of Agriculture at Lyons; of the Medical Society of Aix in Provence; of the Philosophical Society at Philadelphia; and of the American Academy of Arts and Sciences, &c.* — — — 111

XIV. *An Account of the Preparation, Mode of Application, and Effects, of a Liniment recommended by* Roncalli *in the Treatment of scrophulous Tumours. By* Henry Streitt, *Professor of Chirurgical Pathology in the Imperial and Royal Medico-Chirurgical Academy at Vienna. From the Transactions of the Academy.* — — 134

XV. *An Account of the Tabasheer. By* Patrick Russell, *M. D. F. R. S. From the Philosophical Transactions.* — 141

XVI. *Ac-*

Page

XVI. *Account of the Nardus Indica, or Spikenard. By* Gilbert Blane, *M. D. F. R. S. From the ſame Work.* —— 153

XVII. *An Account of a Child with a double Head. By* Everard Home, *Eſq. F. R. S. From the ſame Work.* — — 164

XVIII. *Caſe of a Gun-ſhot Wound in the Mouth; in which, on account of impeded Deglutition, a flexible Catheter was introduced through the Noſe into the Oeſophagus, and ſuffered to remain there during the Space of a Month. By M.* Manoury, *Surgeon of the Hotel Dieu at Paris. From the Journal de Chirurgie.* — 176

XIX. *Account of an extraordinary Change, not hitherto deſcribed, which, under certain Circumſtances, takes place in the human Body after Death. From a Work by M.* Thouret, *entitled 'Rapport ſur les 'Exhumations du Cimetiere et de l'Egliſe 'des Saints Innocens.'* — — 186

*Catalogue of Books.* — — — 201

*Index.* — — — — 217

DIREC-

